# *Mycoplasma pneumoniae* Outbreak at a University — Georgia, 2012

**Published:** 2013-08-02

**Authors:** Melissa Tobin-D’Angelo, Audrey Martyn, Ashley Moore, Hope Dishman, Cherie Drenzek, Kim Turner, Lauri Hicks, Maureen Diaz, Brianna Petrone, Bernard Wolff, Alvaro Benitez, Jonas Winchell, Laura Edison

**Affiliations:** Georgia Dept of Public Health; Fulton County Dept of Health and Wellness; Div of Bacterial Diseases, National Center for Immunizations and Respiratory Diseases; EIS Officer, CDC

On October 17, 2012, the Georgia Department of Public Health (DPH) was notified by the Fulton County Department of Health and Wellness that a local university, the Georgia Institute of Technology, was experiencing a pneumonia outbreak among students. DPH epidemiologists investigated to identify the etiology, find additional cases, and recommend control measures. Respiratory swabs collected from students with pneumonia and tested at CDC using a quantitative real-time polymerase chain reaction (qPCR) assay were positive for *Mycoplasma pneumoniae.* The university alerted students, faculty, and staff members to the outbreak and recommended prevention measures by e-mail, social media, and posters. A survey administered to students assessed illness prevention behaviors, outbreak awareness, and communication preferences. Eighty-three cases were diagnosed among students during September 1–December 4, 2012, making this outbreak the largest reported at a U.S. university in 35 years (1). No cases were reported among faculty or staff members. Of the 83 patients, 19 had specimens tested by qPCR, of which 12 (63%) were positive for *M. pneumoniae*. Despite university communication efforts, approximately half of students surveyed were unaware of the outbreak when surveyed in December. DPH recommendations included implementing university policies that facilitate students staying home and seeking medical care when ill and refining health messages and communication methods to improve awareness of disease outbreaks among students.

*M. pneumoniae* is a common cause of respiratory infection among children and young adults, causing up to 40% of all cases of community-acquired pneumonia (2). In rare cases, *M. pneumoniae* can cause extrapulmonary manifestations, including neurologic, dermatologic, hematologic, and cardiac syndromes. Recommended first-line antibiotic treatments include tetracyclines and macrolides; however, a growing trend of macrolide-resistance worldwide has been reported (2,3). Outbreaks largely occur in closed and semiclosed settings, including schools and universities (4–6).

To investigate the outbreak, university health services records were reviewed weekly, beginning October 17, 2012, to identify cases of pneumonia among students, and information regarding demographics, signs and symptoms, underlying conditions, and treatment was collected. Retrospective record review was performed to identify cases diagnosed as early as September 1, 2012. Beginning on October 17, 2012, oropharyngeal and nasal or nasopharyngeal swabs were obtained from consenting students who agreed to testing and who received a diagnosis of pneumonia from a university health services physician. Initial patient specimens were tested using qPCR for 20 respiratory pathogens to identify the causative agent (7); subsequent specimens were tested for *M. pneumoniae*, *Chlamydophila pneumoniae*, and *Legionella* species using a multiplex qPCR assay (8). Culture and macrolide resistance testing were attempted on all *M. pneumoniae*-positive specimens using previously described methods (9).

Probable cases were defined as a physician diagnosis of pneumonia in a Georgia Institute of Technology student during September 1–December 4, 2012, with or without qPCR evidence. Confirmed cases met the criteria for a probable case and had *M. pneumoniae* detected in oropharyngeal, nasal, or nasopharyngeal swabs by qPCR*.* During September 1–December 4, 2012, a total of 83 cases were identified, including 12 confirmed and 71 probable cases. Illness onset occurred during August 4–December 2, 2012 and peaked at the beginning of November (Figure). Patients were predominantly men (72%), and the age range was 18–30 years (median: 21 years), both representative of the overall student population. Ten (12%) patients had underlying asthma; this rate of asthma is statistically similar to the expected rate among adults aged 18–24 years in Georgia, according to results from the 2010 Behavioral Risk Factor Surveillance System survey.* A total of 79 patients (95%) reported cough, 64 (77%) fever, 36 (43%) headache, and 34 (41%) sore throat. Five (6%) patients with no underlying medical conditions were hospitalized with complications, including four with respiratory failure and one with perimyocarditis; all recovered. Forty-three (52%) patients were treated with doxycycline, 23 (28%) with azithromycin, and seven (8%) with other or multiple antibiotics (Table 1).

Nineteen (23%) of 83 cases had specimens tested by qPCR; 12 (63%) specimens were positive for *M. pneumoniae* and were cultured, yielding 10 isolates identified as *M. pneumoniae*. No other pathogens were identified. All 12 qPCR-positive specimens tested negative for macrolide resistance by qPCR testing of the primary specimen or isolate. Radiographs were administered for 99% of students with pneumonia diagnoses; 61 (74%) had radiographic findings consistent with pneumonia.

## Public Health Response

On November 7, 2012, DPH provided the university with recommendations to curtail the outbreak, including initiation of an outreach campaign to alert the university community to the outbreak and education regarding preventive health behaviors to reduce the spread of illness. During November, the university began an outreach campaign that included a universitywide e-mail, social media postings, and posters displayed around campus. Communications focused on measures to prevent respiratory illness, including proper hand hygiene, respiratory hygiene, and staying home and seeking medical care when ill with a cough and fever.

To assess the effectiveness of the outreach campaign, investigators administered a knowledge, attitudes, and practices survey on December 5, 2012, 2 weeks before winter break, to a convenience sample of 105 students who did not have pneumonia; some students did report having a mild illness during the fall semester. These students were asked to select one or more options from a list to indicate 1) their behaviors whenever they were ill with cough and fever, 2) their preferred health communication methods, and 3) if and how they became aware of the pneumonia outbreak. Of the 105 students surveyed, 48 (46%) were aware of the outbreak; of these 48 students, 38 (79%) reported that they were more likely to use good respiratory hygiene as a result of this knowledge. Thirty-one (30%) of 105 students surveyed indicated that they would stay home with a cough and fever, and 34 (32%) said they would seek medical care for those symptoms. Twenty-seven (26%) students learned about the outbreak from an e-mail, 23 (22%) from a friend, six (6%) from a poster, and two (2%) from social media.

When asked to identify “the best way to communicate these matters to you” and told they could indicate more than one method, 93 students (89%) said they preferred receiving health communications by e-mail, 45 (43%) indicated posters placed on campus, 19 (18%) indicated social media, and 19 (18%) indicated text messages (Table 2).

The last *M. pneumoniae* case was identified on December 4, 2012. Active surveillance was discontinued at university health services on January 1, 2013.

### Editorial Note

The outbreak of *M. pneumoniae* at the Georgia Institute of Technology is the largest reported at a U.S. university in 35 years (1). *M. pneumoniae* has been implicated in other pneumonia outbreaks at universities (5) and is recognized as a common cause of community-acquired pneumonia among university students (4). These outbreaks can be prolonged because of the long incubation period (up to 3 weeks) (2). Because *M. pneumoniae* infection most commonly causes upper respiratory illness (only an estimated 3%–10% of persons with infection experience pneumonia [2,3]), infected persons often go about their normal activities and infect others, as in this outbreak. No cases were identified among faculty or staff members, perhaps in part because they generally do not use university health services. The five hospitalizations demonstrate the risk for severe complications during substantial outbreaks of *M. pneumoniae*; early outbreak recognition is critical because control measures can limit transmission and complications (3).

The multiplex qPCR assay was used as the primary testing method in this investigation because of the documented sensitivity and specificity (8), cost-effectiveness, and status as a Clinical Laboratory Improvement Amendments (CLIA)–approved testing method. Culture, a less reliable method for *M. pneumoniae* detection (2), was used in this investigation to obtain isolates that could be further characterized through additional molecular testing methods.

*M. pneumoniae* is spread through respiratory droplets; therefore, preventive health behaviors, including proper hand and respiratory hygiene and self-isolation when ill, can limit the spread of disease (3). No data are available to support the use of antibiotic prophylaxis during university outbreaks. Inducing preventive health behaviors among university students requires that they become aware of the outbreak, perceive a personal risk, and know of a behavior that can reduce the risk for infection (10). In a survey of 105 students, 54% said they were unaware of the outbreak despite a campuswide e-mail message, social media postings, and posters placed around campus. Among the 46% of students who were aware of the outbreak, 79% said they would be more likely to engage in preventive health behaviors because of this awareness.

To be effective during a *M. pneumoniae* outbreak at a university, health messages need to reach students and educate them regarding their risk for infection and behaviors that can prevent infection. More research is needed to determine the most effective ways to communicate these messages to university students. Sending multiple e-mails and text messages with attention-getting words in the subject line (e.g., outbreak or pneumonia) and the use of informal social networks (e.g., announcements at group activities or in classes) might serve to increase awareness. Effective communications, coupled with university policies that facilitate students staying home and seeking medical care when ill, might reduce transmission of *M. pneumoniae* and the severe complications that can go with it.

What is already known on this topic?

*Mycoplasma pneumoniae* is a common cause of respiratory infection and community-acquired pneumonia among young adults. Approximately 3%–10% of persons infected with *M. pneumoniae* experience pneumonia, and a limited proportion of persons can experience extrapulmonary manifestations, including cardiac syndromes. Outbreaks are known to occur in closed and semiclosed settings, including schools and universities.

What is added by this report?

During September 1–December 4, 2012, a total of 83 cases of *M. pneumoniae* infection were diagnosed among students at a university in Georgia, the largest reported outbreak of *M. pneumoniae* at a U.S. university in 35 years. Despite multiple communication efforts, approximately half of students surveyed in December said they were unaware of the outbreak. Students unaware of an outbreak are unlikely to adopt preventive health behaviors that might limit disease spread

What are the implications for public health practice?

*M. pneumoniae* should be considered in outbreaks of pneumonia among university students. University students are more likely to engage in preventive health behaviors when they are aware of an outbreak, yet students can be difficult to reach, even when their preferred methods of health communication are employed. Health messages should be tailored to reach students and educate them about their risk for infection, and behaviors to prevent infection; more research needs to be done to determine the most effective way to communicate health messages to students.

## Figures and Tables

**FIGURE:**
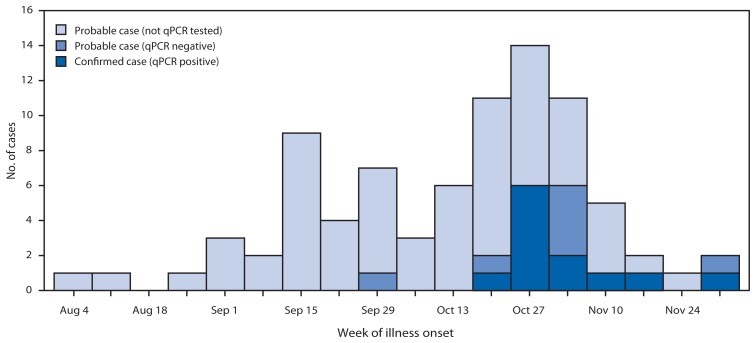
Number of *Mycoplasma pneumoniae* cases (N = 83*) among students at a university, by week of illness onset and qPCR testing status — Georgia, 2012 **Abbreviation:** qPCR = quantitative real-time polymerase chain reaction. * Probable cases were defined as clinically diagnosed pneumonia, with or without qPCR evidence of pneumonia, among students at the university during September 1–December 4, 2012. Confirmed cases were probable cases with *M. pneumoniae* detected in oropharyngeal, nasal, or nasopharyngeal swabs by qPCR.

**TABLE 1:** Number and percentage of *Mycoplasma pneumoniae* cases (N = 83) among students at a university, by selected characteristics — Georgia, 2012

Characteristic	No.*	(%)
**Case type**	83	(100)
Confirmed case	12	(14)
Probable case	71	(86)
**Sex**		
Men	60	(72)
Women	23	(28)
**Symptom**		
Cough	79	(95)
Fever	64	(77)
Shortness of breath	23	(28)
Body aches	26	(31)
Headache	36	(43)
Sore throat	34	(41)
Nasal congestion	32	(39)
Rash	3	(4)
**Radiograph**		
No. of patients administered	82	(99)
No. with findings consistent with pneumonia	61	(73)
**Underlying condition**		
Asthma	10	(12)
Allergies	5	(6)
**Antibiotic treatment**		
Azithromycin	23	(28)
Doxycycline	43	(52)
Other†	7	(8)
None	3	(4)
Information missing	7	(8)

**Abbreviation:** qPCR = quantitative real-time polymerase chain reaction.

* Probable cases were defined as clinically diagnosed pneumonia, with or without qPCR evidence of pneumonia among students at the university during September 1–December 4, 2012. Confirmed cases were probable cases with *M. pneumoniae* detected in oral or nasal or nasopharyngeal swabs by qPCR.

† Includes combination therapy, ceftriaxone, erythromycin, clarithromycin, and levofloxacin.

**TABLE 2:** Knowledge and practices of surveyed students (N = 105) regarding outbreak of *Mycoplasma pneumoniae* at a university — Georgia, 2012

Knowledge/Practices	No.	(%)
**Are you aware of increased pneumonia on campus?**		
Yes	48	(46)
No	57	(54)
**How did you hear about it?**		
E-mail	27	(26)
Friend	23	(22)
Posters around campus	6	(6)
Health services website	5	(5)
Professor	4	(4)
Social media	2	(2)
**Best way to communicate these matters to you?** *		
E-mail	93	(89)
Posters in common areas	45	(43)
Social media	19	(18)
Text messages	19	(18)
Campus newspaper	17	(16)
Residence advisors	15	(14)
Academic advisors	6	(6)
**Which are you most likely to do if you become ill?** *		
Cover cough and sneezes more often	62	(59)
Take over-the-counter medications	53	(50)
Wash hands more	46	(44)
Go to a doctor	34	(32)
Stay home when not feeling well	31	(30)
Do nothing	7	(7)
**Are you more likely to wash your hands, cover your cough, or stay home if ill after seeing the posters and e-mails? (n = 48)**		
Yes	38	(79)
No	10	(21)

* Students could select multiple answers.

## Footnotes

* Additional information available at http://www.cdc.gov/brfss.
